# Levosimendan as Treatment Option in Severe Verapamil Intoxication: A Case Report and Review of the Literature

**DOI:** 10.1155/2010/546904

**Published:** 2010-08-11

**Authors:** Mirjam Osthoff, Christine Bernsmeier, Stephan C. Marsch, Patrick R. Hunziker

**Affiliations:** Medical Intensive Care Unit, University Hospital Basel, 4031 Basel, Switzerland

## Abstract

Cardiovascular shock due to verapamil intoxication is often refractory to standard resuscitation methods. Recommended therapy includes prevention of further absorption of the drug, inotropic therapy, calcium gluconate, and hyperinsulinemia/euglycemia therapy. Often further measures are needed such as ventricular pacing or mechanical circulatory support. Still, mortality remains high. Levosimendan, an inotropic agent, that enhances myofilament response to calcium, increases myocardial contraction and could therefore be beneficial in verapamil intoxication. Here, we report the case of a 60-year-old patient with clinically severe verapamil poisoning who presented with shock, bradycardia, and sopor. Standard therapy including high-dose inotropes failed to ameliorate the signs of intoxication. But additional therapy with levosimendan led to rapid improvement. Based on this observation, the literature is reviewed focusing on utilization of levosimendan in the treatment of calcium channel blocker overdose. We suggest to consider levosimendan as additional treatment option in patients with cardiovascular shock due to verapamil intoxication that are refractory to standard management.

## 1. Introduction

Verapamil antagonizes L-type calcium channels and thereby inhibits calcium influx into myocardial and vascular tissue. The negative inotropic and chronotropic effects of verapamil may result in bradycardia, decreased cardiac output, vasodilation of smooth vascular muscle, and cardiovascular collapse [1]. Calcium channel blockers also inhibit the influx of calcium into pancreatic beta cells. This leads to reduced insulin secretion and hyperglycemia [2]. In consideration of these effects, treatment of calcium channel blocker overdose includes volume application, intravenous calcium to increase calcium availability, inotropic therapy, and hyperinsulinemic euglycemia therapy [3]. Even with extensive conventional treatment, requiring high doses of inotropic therapy [4–11], additional invasive therapies such as pacemaker therapy [5, 9, 10] or IABP insertion [10] are necessary in severe cases to stabilize hemodynamics. However, mortality remains high [12].

Levosimendan, an inotropic agent, acts as a calcium sensitizer and increases the association rate of myosin actin cross-bridges and slows down their dissociation rate by binding to troponin c [13]. Its impact is calcium dependent [13, 14]. Levosimendan also exhibits vasodilation via ATP-sensitive potassium channels in vascular smooth-muscle cells, and recently it also has been shown to act on mitochondrial ATP-sensitive potassium channels [15]. The mechanism is believed to be responsible for its cardioprotective properties in reversing myocardial stunning [15]. The positive inotropic effect of levosimendan seems to be preserved with acidosis [16]. 

Levosimendan has been successfully used in the setting of acute myocardial failure following myocardial infarction with few side effects [17, 18].

Based on these observations, in theory, levosimendan has the potential to be beneficial in the cardiovascular compromise caused by verapamil overdose.

## 2. Case Presentation

Here, we present the case of a 60-year-old male patient that was conducted to hospital with the suspicion of cardiogenic shock.

Paramedics were called for a patient, who had treated himself with 6 tablets of verapamil 80 mg within a period of 6 hours for palpitations. He had a history of intermittent supraventricular tachycardia and therefore had been prescribed verapamil as needed. The patient was conscious, blood pressure was 91/51 mmHg, and the pulse was 46 bpm. The patient received isotonic crystalloids and 1 mg of atropine with no change in heart rate during the transport. Upon arrival at the emergency department, vital signs included blood pressure of 98/79 mmHg, pulse of 41 bpm, respiratory rate of 20/min and temperature of 36.1 degree Celsius. The ECG showed sinus arrest and a wide QRS complex. Immediately, the patient was given isotonic crystalloids, calcium gluconate 10% (1 g) intravenously, and 70 U of insulin in dextrose. A transthoracic echocardiography showed diffuse hypokinesia with a slightly reduced ejection fraction. Blood pressure and pulse declined to 70/32 mmHg and 35 bpm, respectively, while the patient became soporous. A bolus of 50 *μ*g of epinephrine was given, and an epinephrine infusion was started at a rate of 20 *μ*g/min. The patient was admitted to the intensive care unit, where a central venous catheter and a femoral arterial line were inserted. Bradycardia, hypotension, ECG changes, and sopor persisted despite continued calcium gluconate administration, hyperinsulinemia/euglycemia therapy, and inotropic therapy with epinephrine.

Because of primary failure of standard therapy in this clinically severe calcium antagonist intoxication, levosimendan was added to treatment.

**Figure 1:**
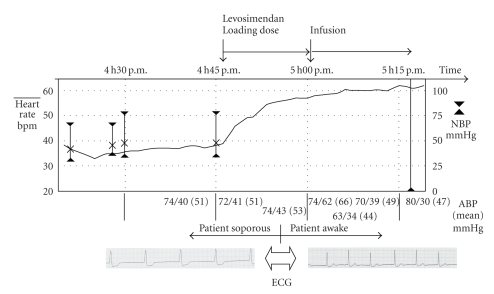
Vital signs in relation to levosimendan application. ABP: arterial blood pressure; NBP: non-invasive blood pressure.

**Figure 2:**
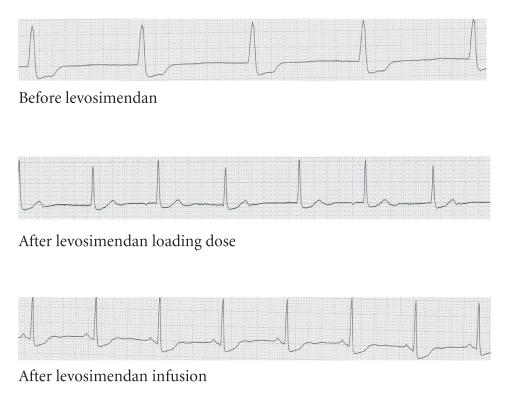
ECG changes in relation to levosimendan adminstration.

A levosimendan loading dose of 24 *μ*g/kg was followed by a levosimendan infusion at a rate of 0.1 *μ*g/kg/h. Within minutes the pulse rate increased steadily to 60 bpm (Figure 1) with reappearance of sinus node activity in the ECG and normalization of the QRS width (Figure 2). The patient regained consciousness but remained hypotensive. Levosimendan was continued for 24 hours. Blood pressure finally stabilized, and epinephrine could be tapered 9 hours after starting levosimendan and stopped after 16 hours. The patient was transferred to the ward after 30 hours and discharged on day 5.

## 3. Discussion

We report a case of verapamil overdose-induced cardiogenic shock. Our patient was resuscitated with conventional treatment including calcium gluconate i.v., hyperinsulinemia/euglycemia therapy, and inotropic therapy with epinephrine according to current recommendations. However, his clinical status, hemodynamics, and ECG alterations did not improve until initiation of levosimendan. Within 15 minutes of infusing the loading dose, the patient regained consciousness, and the pulse rate increased to 60 bpm. Conversion to normofrequent sinus rhythm and stabilisation of blood pressure were achieved during the following 16 hours of continued combined therapy with epinephrine and levosimendan.

In a rodent model of verapamil toxicity levosimendan increased cardiac output; however blood pressure remained low [19]. Hypotension in our patient may have been maintained by levosimendan's agonistic properties on peripheral vascular K(+)(ATP) channels [19].

In another rat model of verapamil toxicity levosimendan did not improve survival; however it was used as a unique antidote [20]. This may be attributed to levosimendan ability to exacerbate the hypotension induced by verapamil intoxication [20].

Although direct evidence is missing, in two reported cases of calcium channel overdose hemodynamics did not improve despite extensive conventional treatment until initiation of levosimendan [10]. 

In the case of our patient, the beneficial effect is quite suggestive. First, the heart rate rose simultaneously with application of levosimendan. Second, the patient regained consciousness, despite prolonged hypotension, presumably due to improved organ perfusion. Last, sinus node activity resumed upon initiation of levosimendan therapy, suggesting a beneficial therapeutic effect on not only the contractile function but also cardiac electrophysiology.

## 4. Conclusions

There is no direct evidence from the literature that levosimendan counteracts the hemodynamic compromise caused by verapamil overdose in humans. However, a beneficial effect is suggestive. In conclusion, levosimendan should be considered as an additional treatment option in verapamil intoxication if standard therapy is insufficient to stabilize hemodynamics.
